# IMG-A1: A Novel Immortalized Granulosa Cell Line for Investigating FSH-Dependent Folliculogenesis and Ovarian Pathophysiology

**DOI:** 10.3390/cells14241940

**Published:** 2025-12-06

**Authors:** Nina M. Alyoshina, Evgenii S. Ruchko, Maria D. Tkachenko, Daria M. Potashnikova, Mikhail A. Lazarev, Yulia O. Nikishina, Mariya S. Vildanova, Ilia I. Zakharov, Viktoria V. Konduktorova, Olga P. Kisurina-Evgenieva, Denis A. Nikishin

**Affiliations:** 1 Department of Embryology, Biological Faculty, Lomonosov Moscow State University, Moscow 119234, Russia; ninalyoshina@gmail.com (N.M.A.); tkmadm@yandex.ru (M.D.T.); mikelazz30@gmail.com (M.A.L.); virgo584@yandex.ru (V.V.K.); 2 Koltzov Institute of Developmental Biology, Moscow 119334, Russia; ruchko@idbras.ru (E.S.R.); zubova.y@gmail.com (Y.O.N.); 3 Department of Cell Biology and Histology, Biological Faculty, Lomonosov Moscow State University, Moscow 119991, Russia; dariapotashnikova@yandex.ru (D.M.P.); vch41048@mail.ru (M.S.V.); work.zakharovilya@gmail.com (I.I.Z.); evgengeva@mail.ru (O.P.K.-E.)

**Keywords:** granulosa cell, in vitro model, FSH receptor, cell immortalization, folliculogenesis, polycystic ovary syndrome (PCOS), aromatase, ovarian follicle

## Abstract

**Highlights:**

**What are the main findings?**

- A stable, non-transformed immortalized murine granulosa cell line (IMG-A1) was developed that maintains a molecular signature characteristic of early antral follicular cells.
- IMG-A1 cells exhibit a physiologically crucial receptor profile (FSHR-positive/LHCGR-negative) and respond functionally to FSH, but not LH/hCG, appropriately modeling early-to-mid follicular development.

**What are the implications of the main findings?**

- The IMG-A1 line overcomes the key limitations of existing models, providing a novel, physiologically faithful in vitro tool for studying FSH-dependent follicular biology.
- This cell line represents a relevant and robust platform for investigating specific cellular responses associated with ovarian disorders and for screening potential therapeutic compounds.

**Abstract:**

The study of ovarian biology is hampered by the lack of in vitro models that faithfully recapitulate the physiology of granulosa cells (GCs). Primary GCs have a limited lifespan, while most immortalized lines are tumor-derived and exhibit non-physiological hormonal responses. The purpose of this study was to develop and characterize a novel immortalized GC line with a stable, physiologically relevant phenotype. We immortalized primary murine GCs from early antral follicles using lentiviral vector to introduce human telomerase reverse transcriptase (hTERT) gene to create the IMG-A1 cell line. The line was extensively characterized using molecular (qRT-PCR, Western blot), cytogenetic (karyotyping), and functional (hormone stimulation, ELISA, proliferation assays) methods to assess its phenotype and responsiveness to gonadotropins and metabolic stressors. Exhibiting a non-transformed phenotype, IMG-A1 cells retain a stable karyotype and express the follicle-stimulating hormone receptor (FSHR) but not the luteinizing hormone/chorionic gonadotropin receptor (LHCGR). Accordingly, they respond to FSH by upregulating steroidogenic genes like aromatase (*Cyp19a1*) but are unresponsive to LH/hCG. Furthermore, the line exhibits physiologically relevant responses to hormonal stimulation, including a strong induction of aromatase by FSH and its synergistic upregulation in a hyperandrogenic and hyperinsulinemic milieu. The IMG-A1 cell line is a unique and robust model of early antral granulosa cells, offering a valuable new tool for studying FSH-dependent folliculogenesis, cellular aspects of ovarian pathophysiology, and drug discovery.

## 1. Introduction

The development of ovarian follicles, or folliculogenesis, is a cornerstone of female reproductive biology, culminating in the release of a competent oocyte for fertilization and the production of hormones essential for regulating the menstrual cycle [1,2]. Central to this intricate process are the granulosa cells (GCs), a dynamic population of somatic cells that proliferate and differentiate in response to endocrine signals, providing structural, metabolic, and signaling support to the growing oocyte [3,4,5]. The precise regulation of GC function, particularly their steroidogenic activity and responsiveness to gonadotropins like follicle-stimulating hormone (FSH) and luteinizing hormone (LH), is critical for fertility [6,7]. Dysregulation of these pathways is implicated in major reproductive disorders, including polycystic ovary syndrome (PCOS) and premature ovarian insufficiency [8].

Studying the molecular mechanisms governing GC biology in vivo is challenging due to the complexity of the systemic endocrine environment and the intricate cell–cell interactions within the ovarian follicle [9,10]. Consequently, in vitro models have become indispensable tools for dissecting specific cellular pathways. The ideal in vitro model of granulosa cells should possess a stable, reproducible phenotype that faithfully recapitulates the key physiological characteristics of their in vivo counterparts [11,12,13]. However, existing models each have significant limitations that compromise their predictive value.

Primary GC cultures, while representing the “gold standard” for physiological relevance, are hampered by a finite lifespan, rapid de-differentiation, and loss of hormonal responsiveness in culture [14,15,16]. Their isolation yields limited cell numbers and is subject to significant batch-to-batch variability, making them unsuitable for long-term, large-scale, or high-throughput screening studies. On the other hand, most currently available immortalized GC lines originate from human or animal ovarian tumors (e.g., KGN, COV434) [17,18,19]. While offering unlimited proliferation, their cancerous origin often results in an aberrant phenotype, including altered signaling pathways, genomic instability, and, most critically, a non-physiological expression profile of gonadotropin receptors. Many of these lines fail to express functional FSH receptors (FSHRs) or aberrantly co-express LH receptors (LHCGRs), preventing the accurate study of FSH-specific signaling, a key event in early-to-mid follicular development [20,21,22,23]. According to the classic ‘two-cell, two-gonadotropin’ model, the acquisition of LHCGR on granulosa cells is a key developmental step that occurs in preovulatory follicles, while early antral follicles are exclusively dependent on FSH. Moreover, recent studies have identified a population of actively proliferating, Protein C Receptor-positive granulosa cells that persist throughout folliculogenesis, driving follicular growth. These cells maintain an early granulosa phenotype characterized by high Fshr and minimal Lhcgr expression [24]. Therefore, a stable cell line that faithfully mimics this FSH-dependent, LH-unresponsive phenotype is critically needed to dissect these specific pathways without the confounding effects of LH signaling.

To bridge this critical gap between the transient nature of primary cells and the non-physiological phenotype of tumor-derived lines, we sought to develop and characterize a novel, immortalized granulosa cell line with high physiological fidelity. By immortalizing primary murine granulosa cells isolated from early antral follicles using human telomerase reverse transcriptase (*hTERT*), we have established a new cell line, designated IMG-A1. By overcoming the major limitations of previous models, the IMG-A1 cell line represents a novel and robust in vitro platform. Its stable, physiologically relevant phenotype makes it an invaluable tool for studying the fundamental mechanisms of FSH-dependent follicular development, for investigating cellular responses associated with endocrine disorders like PCOS, and for screening potential therapeutic compounds, thereby aligning perfectly with the goal of advancing predictive and translational in vitro systems.

## 2. Materials and Methods

### 2.1. Animals and Ethical Statement

All animal procedures were approved by the Institutional Animal Care and Use Committee of the N.K. Koltsov Institute of Developmental Biology, Russian Academy of Sciences (IDB RAS). Female C57BL/6 mice (2 months old) were housed at the Center for Collective Use of Biological Models of IDB RAS under controlled conditions (22–24 °C, 14-h light/10-h dark cycle) with ad libitum access to food and water. To stimulate follicular growth, mice were administered an i.p. injection of 5 IU of Pregnant Mare Serum Gonadotropin (PMSG; Sergon, Bioveta Inc., Ivanovice na Hané, Czech Republic). A total of 3 female mice were used in this study. In this study, animals were used only for granulosa cell isolation; no survival or behavioral experiments were performed. All animal procedures were performed to minimize stress and were conducted in accordance with the Council of the European Communities Directive of 24 November 1986 (86/609/EEC).

### 2.2. Primary Granulosa Cell Isolation and Immortalization

Ovaries were collected 36 h post-PMSG injection to isolate granulosa cells from growing follicles at their most functionally active state. For isolation, ovaries were briefly incubated in a solution of 6 mM EGTA and 0.5 M sucrose [25]. Granulosa cells were then mechanically released by puncturing early antral follicles with a 26.5 G needle in DMEM/F-12 medium. The resulting cell suspension was filtered through a 40-µm nylon cell strainer (BD Biosciences, Franklin Lakes, NJ, USA) to remove oocytes and cell aggregates. Cell viability was assessed by trypan blue exclusion.

Primary granulosa cells were seeded at a density of 0.9 × 10^6^ cells/well in a 6-well plate in Granulosa Medium, consisting of DMEM/F-12 supplemented with 10% Fetal Bovine Serum (FBS), 1× GlutaMAX, 100 nM androstenedione, and 1% Penicillin–Streptomycin (all from Gibco, Thermo Fisher Scientific, Waltham, MA, USA). Immortalization was performed 48 h after seeding as previously described [26]. A third-generation lentiviral vector carrying the hTERT gene was employed. Lentiviral particles were produced in HEK293T packaging cells by transient transfection with the plasmids required for lentiviral assembly—pRSV-Rev (Addgene #12253), pMDLg/pRRE (Addgene #12251), pMD2.G (Addgene #12259)—together with the pBABE-neo-hTERT plasmid (Addgene #1774) [27], which encodes the hTERT transgene. Culture supernatants containing lentiviral particles were collected and stored at –80 °C until use.

Transduction of murine granulosa cells was performed at the first passage following the isolation of the primary culture from C57BL/6 mice. Two days prior to transduction, cells were passaged and cultured until reaching 50–70% confluency. The procedure was carried out in DMEM/F12 medium (PanEco, Moscow, Russia) supplemented with 5% heat-inactivated FBS (Capricorn scientific, Ebsdorfergrund, Germany), 2 mM of L-alanyl-L-glutamine (Thermo Fisher Scientific, Waltham, MA, USA), and 10 µg/mL polybrene (neoFroxx, Hesse, Germany). Granulosa cells were pre-incubated in this transduction medium for 1 h prior to the addition of lentiviral particles. Viral supernatants were thawed on ice at +4 °C, then added to the cells at a 1:1 ratio with the transduction medium. Cells were maintained in a humidified CO_2_ incubator at 37 °C, 5% CO_2_, and the culture medium was replaced 48 h after the addition of lentiviral particles. Puromycin (Gibco, Thermo Fisher Scientific, Waltham, MA, USA) selection was initiated 24 h later at 0.2 µg/mL, and the concentration was gradually increased to a final maintenance dose of 0.8–1.0 µg/mL. Surviving cell colonies were expanded and maintained as the IMG-A1 cell line.

The established IMG-A1 cell line was deposited in the Cell Culture Collection of the N.K. Koltzov Institute of Developmental Biology RAS.

### 2.3. Cell Culture and Treatments

The IMG-A1 cell line and the human granulosa tumor line COV434 (Sigma-Aldrich, St. Louis, MO, USA) were maintained in Granulosa Medium at 37 °C in a humidified 5% CO_2_ atmosphere. Cells were passaged at 80–90% confluency using 0.25% Trypsin-EDTA (PanEco, Moscow, Russia).

For functional experiments, cells were seeded and allowed to adhere for 24 h before treatment. For gonadotropin stimulation, cells were treated for 48 h with 0.1 UE/mL recombinant human FSH (GONAL-f^®^, Merck, Darmstadt, Germany), 0.1 UE/mL human chorionic gonadotropin (Chorulon^®^, Merck & Co., Inc., Rahway, NJ, USA), or 0.1 UE/mL PMSG. For aromatase inhibition, cells were treated for 48 h with 1 µM of letrozole (Sigma-Aldrich, St. Louis, MO, USA). To mimic metabolic dysfunction, cells were treated for 48 h with 100 nM of testosterone (Sigma-Aldrich, St. Louis, MO, USA) alone or in combination with 100 nM of insulin (Sigma-Aldrich, St. Louis, MO, USA). Control cells were incubated in medium containing the corresponding amount of vehicle (DMSO) for the same duration.

### 2.4. Cell Proliferation and Senescence Assays

The proliferation rate was estimated using an MTT assay. Cells were seeded in a 96-well plate at 1 × 10^4^ cells/well and cultured for 1, 2, 3, or 4 days. At each time point, 10 µL of 3 mg/mL MTT (PanEco, Moscow, Russia) was added for 1 h at 37 °C. The medium was then removed, and formazan crystals were dissolved in 60 µL of DMSO (PanEco, Moscow, Russia). Absorbance was measured at 530 nm using a Uniplan microplate reader (Picon, Moscow, Russia). The fraction of proliferating cells was determined by Ki67 immunocytochemistry (see Section 2.8). The incidence of micronuclei was quantified from the same DAPI-stained slides.

Cellular senescence was assessed at passage 18 using the Senescence β-Galactosidase Staining Kit (Cell Signaling Technology, Danvers, MA, USA) according to the manufacturer’s protocol. Cells were fixed, washed, and incubated with the X-Gal staining solution for 5 h at 37 °C. Stained cells were imaged using a Leica DMIL LED microscope (Leica, Wetzlar, Germany).

### 2.5. Cell Cycle and Karyotype Analysis

For cell cycle analysis, cells were harvested, fixed in ice-cold 70% ethanol, and stained in 1 mL PBS containing 25 µg/mL propidium iodide (PI; Carl Roth, Karlsruhe, Germany) and 5 µg/mL RNase A (Thermo Fisher Scientific, Waltham, MA, USA). Mouse blood cells and primary granulosa cells served as a diploid control. Samples were analyzed using a FACSAria SORP cell sorter (BD Biosciences, San Jose, CA, USA).

For karyotype analysis, cells were synchronized by a 24-h incubation with 20% FBS, followed by a 2-h mitotic arrest with 0.1 µg/mL nocodazole (Sigma-Aldrich, St. Louis, MO, USA). Metaphase spreads were prepared following a standard protocol involving hypotonic treatment (0.075 M KCl), fixation in Carnoy’s fixative (3:1 ethanol–acetic acid), and dropping onto cold glass slides. Chromosomes were stained with 4% Giemsa solution and imaged on a Leica DM750 microscope (Leica, Wetzlar, Germany). Chromosome counts were performed using ImageJ software (v1.54p).

### 2.6. RNA Extraction and Quantitative Real-Time PCR (qRT-PCR)

Total RNA was extracted using ExtractRNA reagent (Evrogen, Moscow, Russia) and treated with DNase I (Thermo Fisher Scientific, Waltham, MA, USA). Purified RNA was reverse-transcribed using random hexamers and M-MLV reverse transcriptase (Evrogen). qPCR was performed on a StepOnePlus Real-Time PCR System (Applied Biosystems, Loughborough, UK) using qPCRmix-HS SYBR + HighROX kit (Evrogen, Moscow, Russia). Gene expression levels were normalized to the reference genes *Rps18* and *Tbp* using the 2^−∆Ct^ method. Primer sequences are listed in Table 1.

### 2.7. Western Blot Analysis

Cells were lysed in RIPA buffer supplemented with protease and phosphatase inhibitors (Roche, Basel, Switzerland). Protein concentration was determined using a BCA assay kit (Thermo Fisher Scientific, Waltham, MA, USA). Equal amounts of protein (10–20 µg) were separated by 10% SDS-PAGE and transferred to a 0.45 µm PVDF membrane (Bio-Rad Laboratories, Hercules, CA, USA). Membranes were blocked in 5% BSA in TBST and incubated overnight at 4 °C with primary antibodies (Table 2). After washing, membranes were incubated with HRP-conjugated secondary antibodies (1:20,000) for 1 h. Signals were detected using an ECL kit (Bio-Rad Laboratories, Hercules, CA, USA) and Fusion FX7 Edge V.070 imaging system (Vilber Lourmat, Collégien, France). HSP90 served as the loading control.

### 2.8. Immunocytochemistry (ICC)

Cells grown on glass coverslips were fixed with 4% paraformaldehyde, permeabilized with 0.5% Triton X-100, and blocked with 5% goat serum. Cells were incubated with primary antibodies (Table 2) for 1 h, followed by Alexa Fluor 488-conjugated secondary antibodies (1:1000; Thermo Fisher Scientific, Waltham, MA, USA) for 1 h. Nuclei were counterstained with 0.1 µg/mL DAPI (Sigma-Aldrich, St. Louis, MO, USA) in mounting medium. Images were acquired using an Axiovert 200M fluorescence microscope (Carl Zeiss, Jena, Germany).

### 2.9. Flow Cytometry

For cell surface marker analysis, single-cell suspensions were incubated with fluorophore-conjugated primary antibodies (Table 2) at concentrations recommended by the manufacturer for 15 min at room temperature, washed with PBS, and analyzed using a FACSAria SORP cell sorter (BD Biosciences, San Jose, CA, USA). Data analysis was performed using FACSDiva v.8.0 (BD Biosciences, San Jose, CA, USA) and FlowJo v.X.0.7 software (FlowJo LLC, Ashland, OR, USA).

### 2.10. Estradiol ELISA

Estradiol concentrations in cell culture supernatants were measured using a commercial Estradiol ELISA kit (Immunotech, Moscow, Russia) according to the manufacturer’s instructions.

### 2.11. Statistical Analysis

All experiments were performed in at least three independent biological replicates. Data are presented as mean ± standard error of the mean (SEM). Statistical analysis was performed using GraphPad Prism 8 (GraphPad Software, San Diego, CA, USA). Comparisons between two groups were made using a two-tailed Student’s *t*-test for normally distributed data or the Mann–Whitney U test for non-normally distributed data. Comparisons among multiple groups were performed using one-way ANOVA followed by Tukey’s post hoc test. A *p*-value < 0.05 was considered statistically significant.

## 3. Results

### 3.1. Establishment and Basic Characterization of the IMG-A1 Granulosa Cell Line

**Figure 1:**
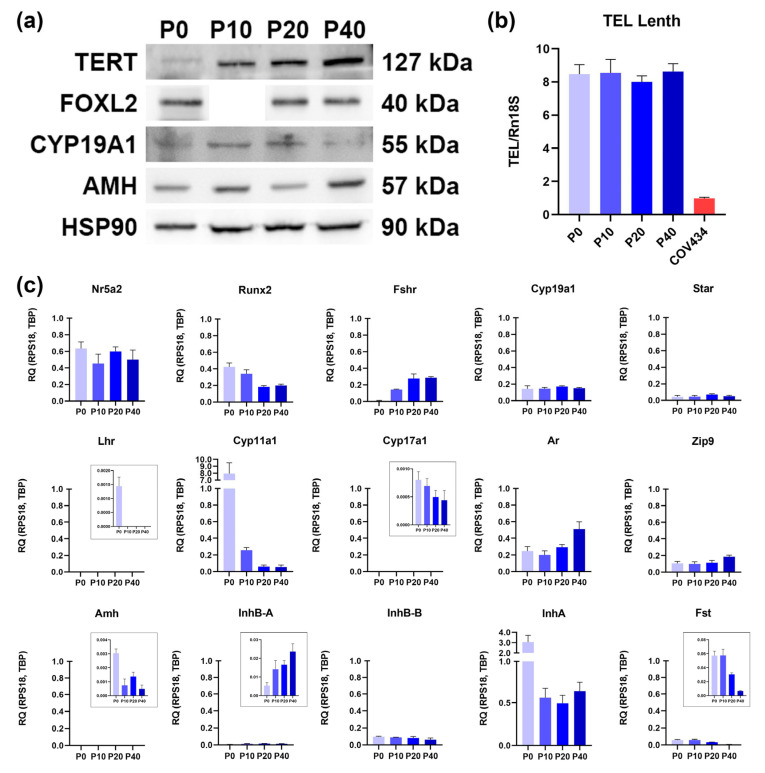
Characterization of marker expression in primary murine granulosa cells (P0) and the immortalized IMG-A1 line at different passages (P10, P20, and P40). (**a**) Western blot analysis of protein levels in cell lysates. HSP90 was used as a loading control. (**b**) Real-time PCR analysis of relative telomere length. Normalization was performed against the multi-copy gene *Rn18s*. The human granulosa cell line COV434 is shown for comparison, demonstrating significantly shorter telomeres than the murine cells. Data are presented as mean ± SEM, *n* = 3 (**c**) qRT-PCR analysis of granulosa cell marker gene expression. Normalization was performed using the reference genes *Rps18* and *Tbp*. Data are presented as mean ± SEM, *n* = 3.

To create a stable in vitro model of granulosa cells, primary murine granulosa cells isolated from growing early antral follicles were immortalized via lentiviral transduction with the human telomerase reverse transcriptase (*hTERT*) gene. The resulting cell line was designated IMG-A1. Successful immortalization was confirmed by the stable expression of the TERT protein for at least 40 passages (Figure 1a) and the maintenance of stable telomere length (Figure 1b).

**Figure 2:**
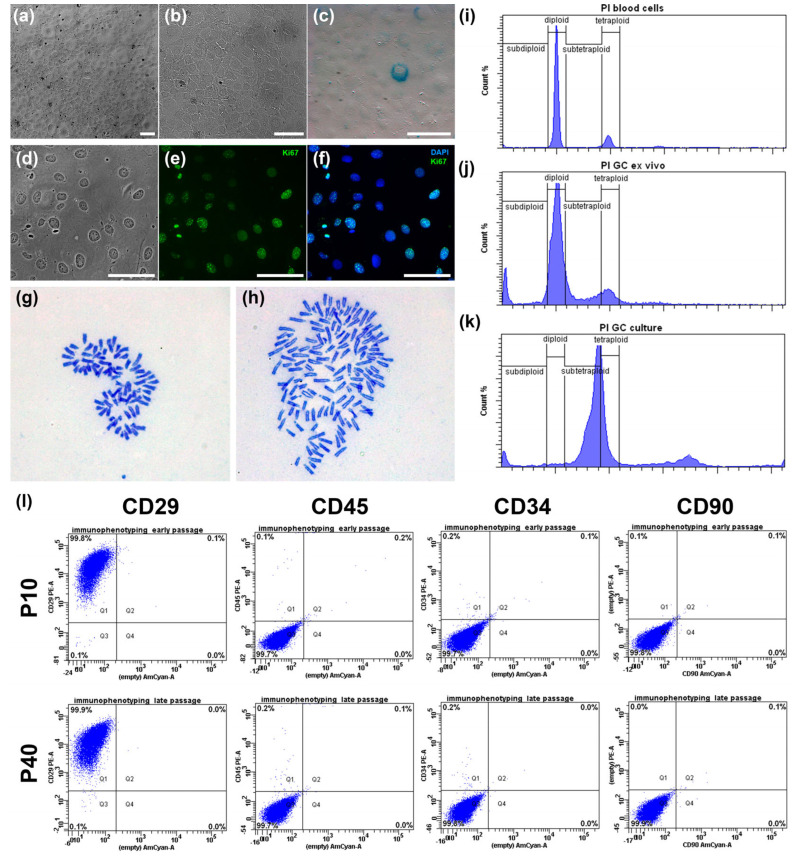
Basic Characterization of the IMG-A1 Granulosa Cell Line. (**a**,**b**) Phase-contrast micrographs of the IMG-A1 cell culture. (**c**) Staining for senescence-associated β-galactosidase activity in passage 18 cells. (**d**–**f**) Immunocytochemical staining for the proliferation marker Ki67, showing a phase-contrast image (**d**), Ki67 immunofluorescence (**e**), and a merged image with DAPI nuclear counterstain (**f**). (**g**,**h**) Representative metaphase spreads showing a normal diploid karyotype (40 chromosomes) (**g**) and a near-tetraploid karyotype (78 chromosomes) from the IMG-A1 line (**h**). (**i**–**k**) Ploidy analysis by flow cytometry. Control blood cells (**i**) and primary granulosa cells (**j**) show characteristic diploid (2n) and tetraploid (4n, G2/M phase) peaks. The IMG-A1 culture (**k**) displays a predominantly near-tetraploid and near-octaploid cell population. (**l**) Flow cytometry analysis confirming the homogeneity of the IMG-A1 culture (passages 10 and 40) based on staining for the stromal marker CD29 and the absence of hematopoietic markers CD45, CD34, and the fibroblast marker CD90. Quadrant gates were set based on unstained controls. Scale bars = 100 µm.

We first characterized the basic morphological and proliferative properties of the line. Under standard culture conditions, IMG-A1 cells displayed a stable and homogeneous epithelioid morphology (Figure 2a,b). At sub-confluent densities, the cells were polygonal and highly flattened, showing strong adherence to the culture plastic. Upon reaching confluence, they formed a classic “cobblestone” monolayer with tight cell-to-cell contacts (Figure 2b). The cells featured a large, prominent nucleus with one or more nucleoli. Importantly, this morphology was consistently maintained for over 90 passages, and no signs of cellular stress, such as vacuolization or spontaneous multilayering, were observed.

The proliferative capacity was high, with a population doubling time of approximately 24 h during the exponential growth phase. This was corroborated by immunocytochemical staining for the proliferation marker Ki67 (Figure 2d–f), which was detected in the nuclei of 92.4 ± 0.4% of the cells, confirming robust mitotic activity.

To assess genomic stability, we analyzed several parameters. The incidence of micronuclei, an indicator of chromosomal damage, was low at 4.0 ± 0.14%. This frequency is substantially lower than that reported for transformed cancer cell lines like A431 (12.6%) and MCF-7 (10.7%), suggesting a relatively stable genome post-immortalization. Furthermore, staining for senescence-associated β-galactosidase (SA-β-gal) revealed that only a minor fraction of the cell population (2.2 ± 0.4%) was positive (Figure 2c). The low percentage of senescent cells indicates that the culture maintains high replicative potential and is not undergoing widespread replicative exhaustion.

Finally, we investigated the ploidy and karyotype of the IMG-A1 line. Flow cytometry analysis (Figure 2i–k) revealed a significant shift in ploidy compared to diploid primary granulosa cells and control mouse blood cells. The IMG-A1 population at passage 20 was predominantly near-tetraploid, with a notable subpopulation of near-octaploid cells (Figure 2k). Karyotype analysis of metaphase spreads confirmed these findings. At passage 18, the cell population was mainly near-tetraploid (77% of cells) with a modal chromosome number of 78 (Figure 2h) (the normal diploid mouse karyotype is 40, Figure 2g). A smaller, near-octaploid population (23%) with a modal chromosome number of 149 was also present. This aneuploid but relatively stable karyotype is a common feature of immortalized cell lines and was consistent between the analyzed passages.

In conclusion, the *hTERT*-immortalized IMG-A1 line is a highly proliferative, non-senescent cell line that maintains the characteristic epithelioid morphology of primary granulosa cells. While immortalization led to aneuploidy, resulting in a stable near-tetraploid karyotype, the cells exhibit a low frequency of chromosomal damage, validating them as a robust and stable model for further functional studies.

### 3.2. IMG-A1 Cells Exhibit a Stable Early Antral Granulosa Cell Phenotype

Flow cytometry analysis of cells at passages 10 and 40 confirmed the high purity and stability of the culture (Figure 2l). The population was highly homogeneous, with 99.8–99.9% of cells positive for the stromal marker CD29. Furthermore, the culture was consistently negative for the hematopoietic markers CD45 (99.7% negative) and CD34 (99.7–99.8% negative), as well as the fibroblast marker CD90 (99.8–99.9% negative), ruling out contamination by other cell types.

To confirm that the IMG-A1 line retains the key molecular characteristics of early antral granulosa cells, we performed a detailed analysis of gene and protein expression, comparing cells at various passages (P10, P20, P40) to primary granulosa cells (P0).

First, we validated the expression of foundational granulosa cell identity markers. Crucially, Western blot analysis confirmed the stable expression of the master transcriptional regulator, FOXL2, across all passages (Figure 1a). Furthermore, the core steroidogenic transcription factors *Nr5a2* and *Runx2* were consistently expressed at the mRNA level, indicating that the fundamental regulatory machinery for granulosa cell function is intact (Figure 1c).

Next, we characterized the steroidogenic profile. The IMG-A1 cells demonstrated a clear specialization towards estrogen production, with high and stable expression of aromatase (*Cyp19a1*) at both the mRNA and protein levels (Figure 1a,c). In stark contrast, the expression of *Cyp11a1*, which encodes the P450scc enzyme characteristic of luteinized cells, was significantly downregulated compared to primary cells. Notably, Cyp17a1 expression was also detected, albeit at a low level, suggesting a limited capacity for endogenous androgen synthesis. Meanwhile, the expression of the cholesterol transporter gene, *Star*, remained at a basal level comparable to that of primary cells (Figure 1c). Collectively, this expression pattern demonstrates that IMG-A1 cells possess a non-luteinized, estrogen-producing phenotype typical of early antral follicles.

A defining feature of the IMG-A1 line is its unique gonadotropin receptor profile. The cells expressed high and stable levels of the FSH receptor (*Fshr*) mRNA, with mRNA levels even exceeding those in primary cultures (Figure 1c). The most critical finding, however, was the complete and consistent absence of LH receptor (*Lhcgr*) mRNA expression across all passages, whereas it was readily detected in the primary cell control (Figure 1c). This unique FSHR+/LHCGR- signature is a hallmark of FSH-dependent granulosa cells prior to final maturation. Crucially, this receptor profile is complemented by the stable, passage-independent expression of both the classical nuclear androgen receptor (*Ar*) and the membrane androgen receptor ZIP9 (*Slc39a9*). The consistent presence of these receptors validates the IMG-A1 line not only as a model for FSH-dependent folliculogenesis but also as a highly relevant platform for investigating the direct effects of androgens on granulosa cell function.

The cells also maintained a complex paracrine signaling profile. Expression of *Inhbb* (encoding the inhibin βB subunit) was consistently higher than *Inhba* (βA subunit), a pattern typical of growing antral follicles (Figure 1c). The cells also stably expressed anti-Müllerian hormone (*Amh*) and the inhibin α-subunit (*Inha*), although *Inha* mRNA levels were an order of magnitude lower than in primary cells, which correlated with protein expression (Figure 1a). Conversely, follistatin (*Fst*) expression showed a tendency to decrease with progressive passaging (Figure 1c).

In conclusion, the detailed molecular characterization confirms that the IMG-A1 cell line stably expresses the molecular markers of early antral granulosa cells. The specific FSHR+/LHCGR-receptor profile, coupled with an estrogen-dominant steroidogenic capacity and a paracrine signature typical of growing follicles, validates IMG-A1 as a highly relevant and specific model for studying FSH-dependent follicular development.

### 3.3. IMG-A1 Cells Demonstrate Selective Functional Responsiveness to FSH

To functionally validate the unique FSHR+/LHCGR- receptor profile identified at the molecular level, we assessed the response of IMG-A1 cells to gonadotropin stimulation. Cells were treated for 48 h with follicle-stimulating hormone (FSH), human chorionic gonadotropin (hCG; an LH analog), or pregnant mare serum gonadotropin (PMSG), which has mixed FSH/LH activity.

**Figure 3:**
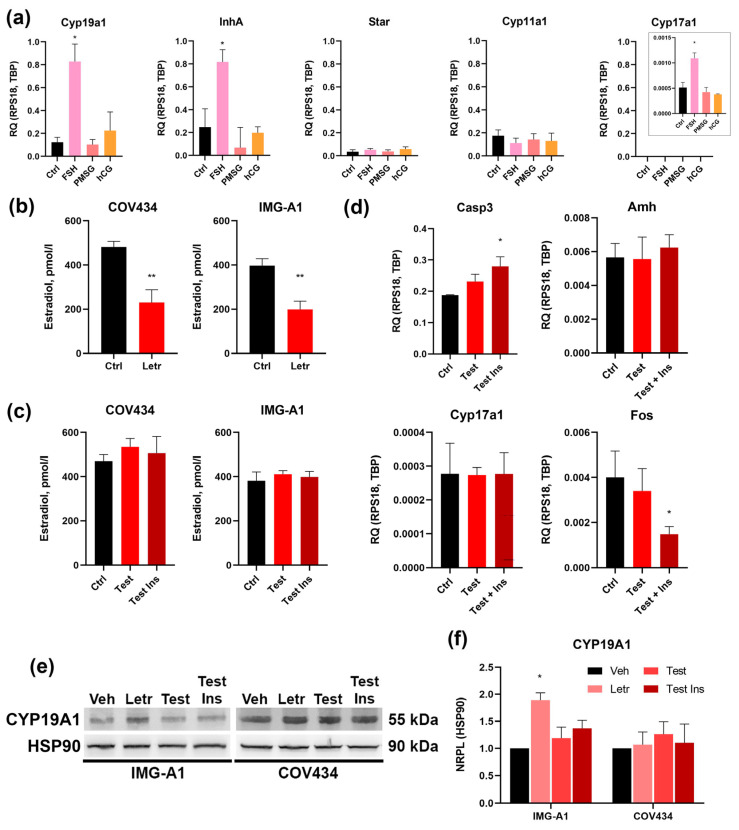
Functional Characterization of IMG-A1 Responsiveness to Hormones and Drugs. (**a**) qRT-PCR analysis of IMG-A1 cell responsiveness to gonadotropic hormones. Data are presented as mean ± SEM, *n* = 3. * *p* < 0.05 compared to the Control group (Kruskal–Wallis test with Dunn’s multiple comparisons test). (**b**) Change in estradiol secretion into the medium in response to letrozole treatment. Data are presented as mean ± SEM, *n* = 3. ** *p* < 0.01 compared to the Control group (Mann–Whitney U test). (**c**,**d**) Analysis of cellular responses to combined testosterone and insulin treatment: (**c**) analysis of aromatase activity via estradiol secretion and (**d**) assessment *Casp3, Amh, Cyp17a1* and *Fos* mRNA expression. Data are presented as mean ± SEM, *n* = 3. * *p* < 0.05 compared to the Control group (Kruskal–Wallis test with Dunn’s multiple comparisons test). (**e**) Western blot analysis of aromatase expression in experiments with letrozole, testosterone, and insulin. HSP90 was used as a loading control. (**f**) Quantification of aromatase protein levels. Data are presented as mean ± SEM, *n* = 3. * *p* < 0.05 compared to the Control group (Kruskal–Wallis test with Dunn’s multiple comparisons test).

As determined by qRT-PCR, stimulation with FSH resulted in a statistically significant upregulation of aromatase (*Cyp19a1*), *Cyp17a1* and inhibin α-subunit (*Inha*) mRNA (Figure 3a). This response is entirely consistent with the physiological action of FSH on granulosa cells in growing antral follicles, which promotes estradiol and inhibin production.

In stark contrast, stimulation with the LH analog hCG had no effect on the expression of these genes, functionally confirming the absence of a responsive LH signaling pathway. Notably, PMSG also failed to induce a significant response at the concentration used. We next examined the expression of the key luteinization markers, *Star* and *Cyp11a1*. Crucially, none of the gonadotropin treatments, including FSH, induced a change in the expression of these genes compared to the untreated control (Figure 3a).

In conclusion, these functional data provide definitive validation of the molecular profile of IMG-A1 cells. The cells exhibit selective responsiveness to FSH, manifested by the upregulation of genes critical for estrogen and inhibin synthesis, while being completely unresponsive to LH/hCG stimulation. The failure of FSH to induce luteinization markers confirms their stable, non-preovulatory phenotype, solidifying their status as a precise model for investigating purely FSH-dependent pathways in ovarian follicular development.

### 3.4. Responsiveness of IMG-A1 Cells to Pharmacological Agents and Hormonal Stimuli

To evaluate the utility of the IMG-A1 line as a tool for studying cellular responses to pharmacological agents and metabolic regulators, we conducted two proof-of-concept experiments modeling key aspects of reproductive physiology and pathology.

To confirm the functional activity of aromatase and explore potential regulatory feedback, IMG-A1 cells were treated with the specific aromatase inhibitor, letrozole (1 µM). As expected, a 48-h treatment caused a sharp decrease in estradiol secretion into the culture medium, as confirmed by ELISA (Figure 3b). Intriguingly, Western blot analysis of cell lysates revealed that this pharmacological inhibition of enzyme activity was accompanied by a significant increase in total aromatase protein levels (Figure 3e,f).

For comparison, the same experiment was performed on the widely used human granulosa tumor cell line, COV434. In these cells, letrozole also effectively reduced estradiol secretion (Figure 3b); however, in stark contrast to IMG-A1, this did not trigger a change in aromatase protein levels (Figure 3e,f). This differential response suggests that IMG-A1 cells possess an intact regulatory feedback loop that attempts to compensate for reduced enzyme activity by upregulating protein synthesis. This physiological-like response is absent in the transformed COV434 line, highlighting the value of IMG-A1 for studying the regulation of the *Cyp19a1* gene.

To investigate the direct effects of androgen and insulin signaling on granulosa cell function, IMG-A1 cells were cultured with testosterone, either alone or in combination with insulin, for 48 h. Treatment with androgens did not alter estradiol secretion (Figure 3c), suggesting that androgen substrate availability is not the rate-limiting factor for aromatase activity under basal conditions. However, significant changes were observed at the molecular level that indicate cellular dysfunction. While exposure to testosterone and insulin induced a notable increase in aromatase protein (Figure 3e,f), suggesting an altered steroidogenic state, a deeper analysis of gene expression revealed a more complex response. Most notably, the combination of testosterone and insulin led to a significant downregulation of the transcription factor *Fos*, a key regulator of granulosa cell proliferation and differentiation. This suppression of a critical developmental driver did not, however, trigger a switch towards androgen production, as *Cyp17a1* expression remained unchanged. Similarly, the expression of *Amh* was not altered, consistent with the understanding that systemic factors or theca-granulosa interactions—absent in this monoculture model—may be required to fully recapitulate the aberrant AMH levels seen in PCOS.

Concurrently with this state of developmental arrest signaled by low *Fos*, we observed a significant increase in cellular stress. The expression of the pro-apoptotic marker *Casp3* was significantly upregulated by pathological stimuli (Figure 3d). Taken together, these data suggest that, in our model, hyperandrogenic/hyperinsulinemic environment does not cause a simple functional shift, but rather induces a state of pathological follicular arrest, characterized by the suppression of key developmental drivers and an increase in apoptotic signaling. These data demonstrate that the IMG-A1 line is responsive to androgen and insulin signaling, resulting in distinct alterations in gene expression and apoptotic markers.

## 4. Discussion

The study of ovarian follicular development has long been constrained by the inherent limitations of available in vitro models. Researchers have faced a difficult choice between physiologically representative but transient primary granulosa cells (GCs) and proliferative but phenotypically aberrant tumor-derived cell lines. In this study, we describe the development and comprehensive characterization of a novel immortalized murine granulosa cell line, IMG-A1, which successfully bridges this gap. Our findings demonstrate that the IMG-A1 line is a stable, non-transformed tool that faithfully recapitulates the key molecular and functional properties of early antral granulosa cells, offering a significant advancement for reproductive biology research.

A primary goal of this work was to achieve immortalization without inducing the transformed, cancerous phenotype that compromises many existing cell lines. By utilizing lentiviral vector to introduce human telomerase reverse transcriptase (hTERT) gene, which primarily addresses replicative senescence, we avoided the widespread and unpredictable cellular changes associated with viral oncogenes [28]. The resulting IMG-A1 line exhibits a stable, near-tetraploid karyotype, consistent proliferation, and minimal signs of senescence or genomic instability over extended culture. This stability is a fundamental prerequisite for a reliable in vitro model, ensuring reproducibility in long-term and high-throughput experimental settings [28].

Our study employed a two-step experimental design, where the IMG-A1 line was first validated against primary cells and then used as the sole platform for functional assays. Primary granulosa cells, while representing the in vivo state, are inherently unstable in culture [14,15,16]. Their tendency to spontaneously luteinize, coupled with a limited lifespan and high inter-experimental variability, makes them an unreliable baseline for the multi-day stimulation protocols used in this study. The IMG-A1 line was specifically developed to circumvent these issues, providing a stable, homogenous, and reproducible system essential for dissecting complex cellular mechanisms.

The defining feature of the IMG-A1 line, and its most significant advantage over other immortalized GC models, is its physiological gonadotropin receptor profile. The cells consistently express high levels of the FSH receptor (*Fshr*) while being entirely devoid of the LH/hCG receptor (*Lhcgr*). This FSHR+/LHCGR- signature is the hallmark of granulosa cells in small to medium antral follicles, which are dependent on FSH for survival, proliferation, and steroidogenic function, but have not yet acquired LH responsiveness [29,30]. Intriguingly, this phenotype also mirrors that of a recently described population of proliferating, Procr-positive granulosa cells that act as progenitors to drive follicular growth throughout folliculogenesis, characterized by a high *Fshr*-to-*Lhcgr* expression ratio [24]. It is therefore plausible that our immortalization and selection process has captured and stabilized this highly proliferative, progenitor-like population, making the IMG-A1 line a unique tool to study the very cells that function as the engine of follicular expansion. Most widely used human GC lines, such as KGN and COV434, either lack FSHR or aberrantly co-express LHCGR, making them unsuitable for dissecting FSH-specific pathways without the confounding effects of LH signaling [31,32]. Our functional data provide definitive validation of this profile: IMG-A1 cells respond robustly to FSH by upregulating key markers of growing antral GC function, including aromatase (*Cyp19a1*) and inhibin alpha (*Inha*), but show no response to LH/hCG. Moreover, even with potent activation of the downstream cAMP pathway, the cells do not induce expression of key luteinization markers like *Star* or *Cyp11a1*. This confirms that the molecular machinery for luteal differentiation is functionally repressed and the cells are locked in a stable, non-preovulatory growing antral phenotype, making them an ideal system for studying purely FSH-dependent follicular dynamics [29]. This is further reinforced by our finding that the initial primary cell population (P0) displayed low levels of *Lhcgr* mRNA, a finding consistent with a heterogeneous starting culture containing a fraction of cells from more advanced follicles. The subsequent establishment of a uniformly *Lhcgr*-negative IMG-A1 line demonstrates the successful purification of the desired FSH-dependent phenotype, resulting in a stable and homogeneous model system that overcomes the inherent variability of primary cultures.

Beyond studying basal physiology, we demonstrated the utility of IMG-A1 for investigating cellular signaling pathways activated by high levels of androgens and insulin [33,34]. A key question is whether the observed cellular changes are truly synergistic or driven by a single component. To address this, we first confirmed the cells’ competence to respond to androgens. Our analysis revealed high mRNA expression of both the classical nuclear androgen receptor *Ar* and the membrane androgen receptor ZIP9 (*Slc39a9*) (Figure 1c), demonstrating that IMG-A1 cells are fully equipped to sense androgens. This finding provides a strong mechanistic basis for our observation that testosterone and insulin act synergistically to upregulate aromatase, confirming the response is not driven by insulin alone. The observed synergistic upregulation of aromatase and the induction of the pro-apoptotic marker *Casp3* resemble key cellular stress responses observed in PCOS follicles [35]. The stark contrast with the unresponsive COV434 line underscores the superior physiological relevance of IMG-A1. Similarly, the compensatory upregulation of aromatase protein following treatment with the inhibitor letrozole suggests the presence of an intact regulatory feedback loop, a feature of a healthy physiological system that is often lost in transformed cells [36].

One of the most insightful findings of our study emerged from the analysis of *Fos* expression, a key component of the AP-1 transcription factor and an immediate early gene tied to cellular proliferation and differentiation [37]. Based on previous work suggesting an inverse relationship between FOS and CYP17A1 in granulosa cells [38], one might hypothesize that the hyperandrogenic, hyperinsulinemic milieu of PCOS would modulate this axis. Strikingly, we observed that the PCOS-like treatment caused a significant downregulation of *Fos* (Figure 3d). However, this was not accompanied by the expected compensatory increase in *Cyp17a1* expression. This suggests that, while IMG-A1 cells respond to these stimuli, they do not undergo the functional switch to an androgen-producing phenotype often seen in theca-interstitial cells or luteinized granulosa cells. The pathological stimulus appears to halt the cells’ normal proliferative and developmental program, a state reflected by the suppressed *Fos* levels. It indicates that, while the cells can sense the “pathological” milieu, they maintain their granulosa cell identity and do not acquire theca-like steroidogenic properties under these specific 2D culture conditions.

Despite its demonstrated utility, it is crucial to acknowledge the inherent limitations of the IMG-A1 model to avoid overinterpretation. First and foremost, the specific FSHR+/LHCGR- profile, while constituting the model’s primary strength for studying early folliculogenesis, inherently limits its application to later stages of development. The complete absence of LHCGR expression means the line cannot model the LH surge, ovulation, or spontaneous luteinization. Second, as a 2D monoculture, the system lacks the complex three-dimensional architecture and the vital paracrine crosstalk with theca cells and the oocyte [39,40]. This limitation is clearly illustrated by our data showing that AMH and androgen pathway gene expression did not mimic the classic PCOS profile in response to hormonal stimulation. This indicates that the hyperandrogenic/hyperinsulinemic milieu alone is insufficient to recapitulate the full systemic pathophysiology of PCOS in this model, suggesting that additional factors—such as theca-derived signals or systemic metabolic inputs—are required. Finally, as a clonal murine line, potential species-specific differences in hormone metabolism must be considered [41], and the clonal nature does not capture the genetic heterogeneity present in the clinical population.

These limitations, however, define clear and exciting avenues for future research. The logical next step is the development of 3D co-culture systems, integrating IMG-A1 with theca cells and oocyte-like structures to reconstitute the follicular microenvironment. Moreover, the stable nature of IMG-A1 makes it an ideal platform for high-throughput screening, for instance, to test libraries of compounds for their potential as endocrine disruptors or to identify novel therapeutic targets. Finally, the application of CRISPR-Cas9 gene editing to this line could generate new models for studying monogenic ovarian disorders, such as premature ovarian insufficiency, opening up new frontiers in reproductive biology research.

## 5. Conclusions

In conclusion, the IMG-A1 cell line addresses significant challenges that have hindered in vitro research in ovarian biology. By providing a stable, reproducible, and physiologically relevant model of an early antral granulosa cell that responds specifically to FSH, we offer the scientific community a valuable new tool. While it does not recapitulate the full complexity of ovarian physiology or systemic disease, this work represents a significant contribution to the field, providing a robust platform for dissecting cell-autonomous mechanisms of reproductive health, disease, and therapeutics.

## Figures and Tables

**Table 1:** Primer sequences used for qPCR analysis.

Gene Name	NCBI Gene ID	Forward Primer	Reverse Primer
*Amh*	11705	CACCCGCTACCTGGTGTTAG	GTACTCAGCCGGGAGTCCT
*Ar*	11835	GAGAGAGGCAGCTTGTGCAT	CGAGACTTGTGCATGCGGTA
*Casp3*	12367	AGATACCGGTGGAGGCTGAC	TTAACGCGAGTGAGAATGTGC
*Cyp11a1*	13070	GCCTGGAGCCATCAAGAACT	GAAAAGCGGAATAGGTCATCACT
*Cyp17a1*	13074	CGGTGGCCCCCTTGCTCA	GGCTGGTCCCATTCATTTTTATCGTG
*Cyp19a1*	13075	TCTCCTCATCAAACCAAACATCTTCT	CAGTTGCAAAATCCATACAGTCTTCC
*Fos*	14281	GGAGAATCCGAAGGGAACGG	AAGGTCATCGGGGATCTTGC
*Fshr*	14309	TGCTACACCCACATCTACCTCACA	GGATCTTGGCCTTGGACACAGT
*Fst*	14313	GCCTGCTGGGCAGATCTATT	TGGATATCTTCACAGGACTTTGCT
*Inha*	16322	TTGTTCTGGCCAAGGTGAGG	AGCTGACTTGTCCTCACAGC
*Inhba*	16323	AGCTCAGACAGCTCTTACCAC	CCTGGCAGCAAAAGTTGTTGT
*Inhbb*	16324	GCGCGTTTCCGAAATCATCA	GGACGTAGGGCAGGAGTTTC
*Lhr*	16867	CTCTCACCTATCTCCCTGTCAAAGTAA	TGTAAAAGCACCGGGTTCAATGT
*Nr5a2*	26424	CCAAGGCCACGAAATTTGACA	ACCAATAGGTGTAAGTCCGTGC
*Rn18s*	19791	GACTCAACACGGGAAACCTCA	CAAATCGCTCCACCAACTAAGA
*Rps18*	20084	AAGAAAATTCGAGCCCATAGAGG	TAACAGCAAAGGCCCAGAGACT
*Runx2*	12393	AATCTCCGCAGGTCACTACC	ACTGTGCTGAAGAGGCTGTTT
*Star*	20845	GCCCACTTTTCTGTCCCTTAT	CTGCCCTCGCTCACCTTA
*Tel*	-	CGGTTTGTTTGGGTTTGGGTTTGG GTTTGGGTTTGGGTT	GGCTTGCCTTACCCTTACCCTT ACCCTTACCCTTACCCT
*Tbp*	21374	GTAGCGGTGGCGGGTATCT	CGTCTTCAATGTTCTGGGTTATCT
*Zip9 (Slc39a9)*	328133	CAGCATTTGGGCTGGTTTCC	CACTCCAGTGGCATTGACCT

**Table 2:** List of antibodies and key reagents for Immunocytochemistry, Flow Cytometry and Western blotting.

Antibody	Manufacturer	Catalogue Number
PE CD29 (Integrin beta 1) Armenian hamster mAb [HMb1-1]	Thermo Fisher Scientific, Waltham, MA, USA	12-0291-82
PE CD34 Rat mAb [RAM34]	Elabscience Biotechnology Co., Ltd., Wuhan, China	E-AB-F1284D
PE CD45 Rat mAb [30-F11]	BD Biosciences, San Jose, CA, USA	553081
BV 510 CD90.2 (Thy1.2) Rat mAb	BioLegend, San Diego, CA, USA	105335
Ki67 Rabbit pAb	Servicebio Technology, Wuhan, China	GB111141
ABflo^®^ 488-conjugated Goat anti-Rabbit IgG (H + L)	ABClonal, Wuhan, China	AS053
Aromatase (CYP19A1) Rabbit pAb	ABClonal, Wuhan, China	A12684
TERT Rabbit mAb	ABClonal, Wuhan, China	A4774
FOXL2 Rabbit pAb	ABClonal, Wuhan, China	A16244
AMH Rabbit pAb	Thermo Fisher Scientific, Waltham, MA, USA	PA5-35851
HSP90 Rabbit pAb	Merck KGaA, Darmstadt, Germany	SAB4300541
HRP goat anti-rabbit IgG	Jackson ImmunoResearch Labs, West Grove, PA, USA	111-035-144

## Data Availability

The original contributions presented in this study are included in the article. The IMG-A1 cell line established in this study is available for researchers at the Cell Culture Collection of the N.K. Koltzov Institute of Developmental Biology, RAS. Further inquiries can be directed to the corresponding author.

## Abbreviations

The following abbreviations are used in this manuscript:

Amh	anti-Müllerian hormone
Casp3	Caspase-3
Cyp11a1	P450scc enzyme
Cyp19a1	aromatase
FBS	Fetal Bovine Serum
FSH	follicle-stimulating hormone
FSHR	follicle-stimulating hormone receptor
Fshr	FSH receptor
Fst	follistatin
GCs	granulosa cells
hCG	human chorionic gonadotropin
hTERT	human telomerase reverse transcriptase
i.p.	intraperitoneal
IMG-A1	immortalized murine granulosa cell line
Inha	inhibin α-subunit
Inhba	inhibin βA subunit
Inhbb	inhibin βB subunit
LH	luteinizing hormone
LHCGR	luteinizing hormone/chorionic gonadotropin receptor
PI	propidium iodide
PMSG	Pregnant Mare Serum Gonadotropin
PCOS	polycystic ovary syndrome
qRT-PCR	Quantitative Real-Time PCR
SA-β-gal	senescence-associated β-galactosidase
SEM	standard error of the mean
Star	cholesterol transporter gene
